# Transactions of the American Medical Association

**Published:** 1855-02

**Authors:** 


### BOOK NOTICES.

### *Transactions of the American Medical Association, Vol. VII.*

In a previous No. of the *Journal*, we acknowledged the receipt of this volume, and promised a more extended notice of its contents.

The address of Dr. Parsons is short, but appropriate to the occasion. It alludes to the future of the Mississippi Valley as the grand theatre of human progress, and thus remarks:

And in no department of human affairs is progress here more sure than in medical knowledge. Our Atlantic States have inherited a reverence for European opinions, which, although commendable in our early medical history, is at the present day less favorable to American progress and discovery in medicine. We need to interrogate nature and experience more, and European opinions less. We need mental as well as political independence, a freer swing of thought and purpose that characterizes our brethren of the West, and which this Association is adapted to call into action.

There is much to encourage you in your recent discoveries and contributions, in the results of the vivisections of saurians, the half of which, if confirmed by future experiments, will shed new light on physiology; and again, in the discoveries made relating to the process of digestion by your late lamented Beaumont, of St. Louis, who, for the theories and speculations before prevailing, has substituted ocular demonstration of the modus operandi of that wonderful process, by submitting to it the various articles of human aliment, and determining the length of time required for converting each into healthful chyme; and again, in the successful labors of Drake in traveling from State to State throughout the valley, collecting the history and character of its epidemics by personal inquiry and observation. Others of your venerated dead might be mentioned, who have pursued a like independent course untrammeled by European authorities. Of their immediate successors who now stand at the head of their profession, it would ill become me to speak, seeing that some of them are present and unused to such freedom of remark But to the junior members of the profession we would say, 'Unite with us—follow the example of the distinguished pioneers I have named, and of Caldwell and Harrison, who have gone to their reward; throw the results of your labors into the common stock of medical knowledge accumulated by this Association, where rest assured that they will be duly appreciated to the common benefit of the profession, and of mankind, and redound eventually to your everlasting honor and professional fame.''

These paragraphs are suggestive of the duty which western physicians owe to the profession and to themselves. It is here in the great central region of the continent that the national character is to be developed, that American literature is to assume its distinctive features, and scientific research be directed to specific objects. This is no vain boast. The unparalleled growth of the past clearly indicates it, the physical geography of the region makes it certain. The Mississippi river, extending from the gulf to the extreme north, with its tributaries, embracing the Alleghany and the Rocky Mountains, is a huge giant, folding in its arms the wide prairies teeming with the most luxuriant vegetation, the fairest maiden that the rays of the sun have ever warmed into life and beauty. From the Alleghany to the Rocky Mountains, the whole country is a unit; although vast in extent, the facilities for communication even now are easy and abundant, and will be still more so. This fact, in connection with the natural resources of the country, must develope here the highest American civilization and the truest and noblest progress in science; and to our hands, as Western physicians, are committed, in a great measure, at least those influences which, though now feeble, must ultimately give character to the American Medical profession. While we acknowledge this responsibility, and regret that we are so poorly prepared to meet it, we point with pride to what has been done for the advancement of our science, even in the infancy of our professional life, and hail it as an earnest of what shall be accomplished in the strength of our manhood.

The next paper is the report of the committee on medical education, furnished by Dr. J. L. Cabell, the chairman. The committee in this report have confined their attention to such measures, prospective or established, in reference to medical education, and the reputable standing of the profession as they have deemed worthy of special consideration, and which are embodied in the following resolutions:

1. *Resolved*, That the views and recommendations heretofore expressed by this Association, respecting the importance of establishing a uniform standard of preliminary education, of extending the terms of lectures, and especially of greatly elevating the standard of professional attainments requisite to graduation, be hereby reaffirmed.

2. *Resolved*, That this Association approves and recommends the practice of daily examinations by each professor, as essential for securing "that active, practical discipline" of the mind which is one of the most important ends of collegiate instructiou; and believes, not only that such a system might be easily put into operation, under an extension of the terms of lectures, but that the whole ground-work of elementary medical instruction might be most aduahtageously assigned to the schools which may adopt that system, as a substitute for the very faulty one of private office instruction now in common use.

3, *Resolved*, That this Association cordially approves of the establishment of "private schools" duly organized for giving that species of instruction which consists "in demonstrations" and other practical exercises "on the part of the student, instead of the instructor, but still under his direction and superintendence," embracing the whole circle of clinical observation and practice, the use of the microscope, chemical manipulations, and the performance of surgical operations on the dead body; and would earnestly recommend such institutions to the patronage of those graduates who did not enjoy similar advantages during the period of their collegiate pupilage.

4. *Resolved*, That those medical colleges whose curriculum does not now include full courses of lectures on physiology and medical jurisprudence, be earnestly invited to make immediate provision for supplying the deficiency, and to require the professor of physiology to make an exposition of the outlines of comparative anatomy, to such extent, at least, as may be necessary to enable the student to appreciate the force of the evidence upon which the modern doctrines of physiology mainly rest.

5. *Resolved*, That to insure the efficient and beneficial operation of the proposed measures of reform, the Association considers it essential that some uniform system of examining candidates for admission into the ranks of the medical profession, *in addition to the collegiate examinations for degrees*, should be adopted in all the States of the Union.

6. *Resolved*, That the Association regards as auspicious omens of future progress the already improved character of our medical literature, and the evidence of an increasing desire, on the part of a respectable number of medical students, for a higher grade of professional education, as exhibited in the patronage extended to extra-collegiate organizations for practical teaching; and that, in view of such encouraging signs, it cherishes an abiding conviction that more thorough and general reforms will be ultimately, though gradually, accomplished.

In reference to the first resolution the convention held at Philadelphia in May, 1847, adopted the following standard as one which might reasonably be expected of every young man who should apply to be received as a student of medicine, "a good English education; a knowledge of natural philosophy, and the elementary mathematical sciences, including geometry and algebra, and such an acquaintance at least with the Latin and Greek languages, as will enable the student to appreciate the technical language of medicine, and read and write prescriptions."

This is certainly a reasonable requisition, but we cannot hope in the present condition of the profession that it will universally or even generally be adopted We cannot expect that physicians will require a higher standard of their pupils so far as preliminary education is concerned, than that to which they themselves have attained. The standard of professional knowledge and ability in the other professions, is regulated by the demands of the public. Such ought to be the case in medicine, and such would be were it not so difficult for the public to judge of the real capacities and attainments of the physicians. The advantages of a thorough course of mental training, combining the study of the languages of science with the severer discipline of the mathematics, as preparations to professional study can hardly be appreciated by those who have not enjoyed in them. Why, then, have not our colleges whose faculties may be supposed to understand these matters, placed the proper sentinels at the entrance of our threshold, thereby preventing the sacredness of our temple from being profaned by disgraceful ignorance? It might require a sacrifice; it probably would for a season, but the honor of a noble profession and the interests of humanity are at stake.

We would not be understood as acknowledging that medical men in this country are lacking in natural ability, or even in independence of thought. On the contrary we are perfectly certain that the ranks of our profession are constantly being filled up by young men of superior qualifications, many of whom in time, notwithstanding the deficiency of their preparatory studies, attain to an honorable distinction among us, for their cultivation of the science and their skill in the art of medicine. But as a body we are not what we should be—not what we are capable of being. The recommendations of the American Medical Association so far, have been almost a nullity, and for the simple reason, as we believe, that public opinion both in and out of the professional ranks, has not yet raised the standard of attainment by which, as physicians, we are to be tried and judged.

In view of the little that has been done towards correcting this evil, the committee enquire—

Is there, then, no remedy for an evil which has done so much to dishonor a profession once noted for the profound and varied learning of its members? Your committee are of opinion that it would be premature to come to so discouraging a conclusion. They do not yet abandon the position which was ably sustained by the late Dr. Parrish, in a report made to the Philadelphia Convention, that "the influence of combined and harmonious action, directed to a special object, by the great body of the profession, is a power more potent than that exercised by legislatures, or by the corporations they may create." The operation of such an influence is, however slow, and we should not be impatient if ample results are not rapidly attained. One of the instrumentalities through which it operates is gradually being brought to bear upon the solution of the difficulties which thwart the attempts that have hitherto been made to introduce this and other measures of medical reform. We allude to the organization of State and County Medical Societies, the manifold usages of which agencies are too obvious to require exposition.

The question of the propriety of lengthening the term of lectures in our colleges is discussed at length by the committee. This involves the question of teaching by private instruction mainly, or in the college and hospital. We cannot forbear quoting that portion of report relating especially to this subject. After alluding to the recommendation of the association at previous meeting, that the lecture term be increased to six months instead of four, they say:

A few of the schools promptly complied with this recommendation, but a part of them, as we are informed, have since it found it expedient to abandon the experiment, and to resume their former practice. This fact illustrates the difficulty attending every attempt to carry out any very decided measure of reform, on the part of a few of the schools, while the majority refuse to co-operate. Unfortunately, there exists a difference of opinion as to the advantages of the proposed extension of the term. It will be remembered that the Medical Faculty of Harvard University, presented to the Association, at its second annual meeting, held in Boston in 1849, a formal defense and advocacy of the four month's course, in preference to a more extended term. The faculty of the Medical Department of Pennsylvania Colleges, in replying to a circular of the Committee on Medical Education for the iame year, appear to have taken a similar view. They assume that the "American System" of medical education consists in the instruction derived from "private preceptorship mainly," with the college and hospital as its complement;" and argue that "either we should labor to perfect that system according to its idea, or we should abandon it for another as radically wrong. Any attempt to alter or amend it, according to an essentially distinct plan, will prove a ruinous patching" They further express the "fear that the recent so-called reform agitation tends to loosen the connection of the preceptor with his pupil, and to lessen his responsibility without adequately supplying the loss;" and contend that "such must be the effect of any considerable increase in the course of lectures." The Committee do not deny that there is some plausibility in this view of the subject, and that some of the arguments advanced have force in their application to the prevalent system of medical education in this country; but they are far from acquiescing in the conclusion that no reform in this respect is needed. They would rather adopt the alternative presented in the argument last cited, and conclude a system which regards the plan for instruction by public lectures "as subordinate and subsidiary" to private instruction, "as radically wrong." This system takes it for granted that even the first-course students have "read" with a private teacher, prior to their attendance on lectures. But when it is borne in mind that the fundamental branches of medical science, on which the whole superstructure of medical education must rest, are precisely those which require, for their proper and successful exposition, the use of such appliances of demonstration as can be supplied by public institutions alone, it will be admitted that such preparatory "reading" is not always advantageous, and may be are termed "college clinics." But even where no other impediment hinders the students from visiting the hospitals daily, those who attend six lectures each day in the college, and are also engaged in the study of practical anatomy in the dissecting room, can find little time or disposition for such clinical observations as will be of any value to them. Hence, it is only the small minority who remain in the city during the summer and autumn that truly derive any profit from the facilities for clinical observation which the city hospitals may afford.

'We have almost invariably found that, among even this class of students, only those who have previously graduated can be induced to read, in connection with their clinical observations, the class of books which constitute the elucidators of disease. The undergraduates feel themselves constrained, by the prospect of a coming examination, to confine their reading mainly to the elementary text-books. In view of such facts, your Committee express their hearty concurrence in the position taken by Dr. Pitcher, in his report, and indorsed by the vote of the Association,—"That a familiar knowledge of the elements of medical science should precede clinical instruction." This course is practically adopted by a large number of the better class of medical students, who pursue clinical studies for a period of from one to three years after graduation, either in the hospitals of our own cities, or partly here and partly in Europe. Having dissolved their connection with the schools, in which elementary instruction alone can be adequately given, and in which the second-course students pursue precisely the same studies as those who are attending lectures for the first time, they properly lay aside elementary books, and substitute, in addition to systematic "Clinical Guides," the published records of the clinical observations of those careful and reliable observers who have done so much towards giving precision and certainty to modern pathological doctrines, by authenticating the data upon which they are based, and clearing away the immense rubbish of "false facts" which, as much as aught else, had retarded the progress of medicine as a positive science. By the aid of such accurate clinical reports, carefully studied in connection with the actual observations of cases at the bedside, the intelligent and earnest pupil may add to his own personal experience that of all the eminent clinical reporters whose works he may read. The descriptions there given of the daily progress of the symptoms, and the effects of remedies, can scarcely be appreciated without the aid derived from an examination of cases of the actual disease; but with this aid, that familiar law of our mental organization in accordance with which vividness is imparted to our conceptions by being associated with sensible objects, is brought into operation, and the reported cases rise before the mental vision with the clearness and distinctness of reality.

tary principles of the medical sciences. In this view of the subject, the Committee are inclined to believe that very great advantage would result from an extension of the course of lectures to even a longer term than that heretofore recommended by the Association. In addition to the very obvious evil of crowding six or seven lectures into the compass of a single day, another striking objection to the present system lies in the fact that the practical branches of medical science are of necessity unfolded to the student before he has had an opportunity of becoming acquainted with the elementary principles upon which they are mainly based. For example, his attention is directed to, and his mind bewildered with, an intricate account of the causes and varieties of convulsive diseases, before he has obtained any useful degree of knowledge of the anatomy and physiology of the nervous system, upon which the prevalent doctrines as to the pathology and rational therapeutical indications of such diseases entirely rest. If the term of lectures were extended, it would be possible to diminish the number of lectures for each day, and to make such a distribution of the several branches as would insure an opportunity for the thorough study of the anatomy and physiology of every organ, before the attention of the student should be directed to its pathological states, while several hours would be appropriated to private reading and reflection, and to the practical study of anatomy by means of dissections by daylight.

Regarding, then, "the American System" of medical education by "private preceptorship mainly," at least as that system is carried out in the rural districts, and consequently, for a large majority of American students, "as radically wrong," your Committee would advocate the substitution of a plan which would bring the whole circle of elementary medical education within the province of the schools."

We have only space to refer to that part of the report in which the value of clinical instruction is discussed. We extract it entire:

"4. The profession objects, with much reason, to the entire neglect or very imperfect use of clinical instruction in many of the medical colleges of the United States. As respects some of these institutions, the defect is irremediable, owing to their location in villages too small to furnish subjects for hospital practice. And in some of our larger cities, the seats of the oldest medical colleges in the Union, the hospitals are either entirely inaccessible to the student, or are opened to him under such restrictions as to make it a privilege of little value In such cases, the schools have been driven to the necessity of finding a substitute in what injurious. A medical student, reading in the office of a physician residing in a rural district, cannot enjoy the advantage of studying practical anatomy. Hence, most of his reading is on the subjects of practical medicine and surgery, in which he forms the most vague and erroneous notions with regard to the structure and laws of action of the organs, the diseases of which he is studying; and such notions do not always easily give place to the simple truth, when this is subsequently presented. In cities and the larger towns, the case is admitted to be often different. There, the pnpils may have access to hospitals and dissecting rooms, and there, too, organized voluntary associations of private teachers supersede, almost wholly, the old system of instruction by a single teacher. Such associations are, however, medical schools in all but the name, and thus do not, in point of fact, constitute a real exception to the principle on which we would insist. Not being entitled to confer the diploma, and basing their claims on the confidence and patronage of the more aspiring students, or the thoroughness of the instruction they offer, many of them give a more full, and especially a more practical course than is required in the regularly incorporated medical colleges. Such private schools are admitted to be most valuable auxiliaries in the cause of medical education, and are entitled, in the opinion of the Committee, to an encouraging notice from the Association. It would, however, be wise, in view both of a judicious economy, and of the likelihood of thereby insuring the largest amount of practical benefit from the educational instrumentalities at the command of the American medical student, to assign to these auxiliary organizations such work only as cannot be adequately performed by the incorporated schools with larger classes,—"such as demonstrations on the part of the student, instead of the instructor, but still under his superintendence and direction;" the actual performance of surgical operations on the dead body; chemical manipulations; the use of the microscope; and clinical instruction, truly so called, which is the study of diseases at the bedside, cases being watched from commencement to end, with special attention to the use of the physical means of diagnosis in diseases of the lungs and heart. Such instruction as this is of inestimable importance. It cannot be given by lectures alone. The student must practice for himself, under the supervision and direction of a competent teacher; aud only a class of limited number can receive adequate attention from one instructor. Such instruction should, therefore, be the appropriate work of a corps of teachers auxiliary to the public schools. From its very nature it can be profitable only to advanced students. In the opinion of your Committee, it should follow, rather than precede or accompany, the instruction imparted in the public schools. The latter should consist mainly in a didactic exposition of the elemen-

"In immediate connection with this topic, the Committee would again invite the attention of the Association to the importance of other branches of medical science which may be studied practically. We may specify practical chemistry, the use of the microscope as an instrument of research, and as furnishing data upon which a differential diagnosis may be based, practical midwifery, and operative surgery. It is a fact which may well elicit our mutual congratulation, that efficient instrumentalities for thorough practical instruction in all these branches, are being brought into operation in various parts of the Union. The advertisement of "the New York Preparatory School of Medicine" is now before us. Such associations, if they fulfill their promise, will deserve the encouragement of the profession. We think, however, that the use of the term "preparatory" may mislead the public, to the detriment, perhaps, of these auxiliayr schools. All instruction received by a pupil is preparatory to the great business of life; but in no sense can such instruction as these schools propose to give, be considered preparatory for that of the incorporated schools. The latter is elementary, and should precede the practical. We shall presently consider, under another head, the objection that may be raised on the score of the difficulty of inducing many students to apply for such instruction, after having received the diploma which entitles them to practice medicine and surgery."

